# Genetic interaction analysis among oncogenesis-related genes revealed novel genes and networks in lung cancer development

**DOI:** 10.18632/oncotarget.26678

**Published:** 2019-03-05

**Authors:** Yafang Li, Xiangjun Xiao, Yohan Bossé, Olga Gorlova, Ivan Gorlov, Younghun Han, Jinyoung Byun, Natasha Leighl, Jakob S. Johansen, Matt Barnett, Chu Chen, Gary Goodman, Angela Cox, Fiona Taylor, Penella Woll, H. Erich Wichmann, Judith Manz, Thomas Muley, Angela Risch, Albert Rosenberger, Jiali Han, Katherine Siminovitch, Susanne M. Arnold, Eric B. Haura, Ciprian Bolca, Ivana Holcatova, Vladimir Janout, Milica Kontic, Jolanta Lissowska, Anush Mukeria, Simona Ognjanovic, Tadeusz M. Orlowski, Ghislaine Scelo, Beata Swiatkowska, David Zaridze, Per Bakke, Vidar Skaug, Shanbeh Zienolddiny, Eric J. Duell, Lesley M. Butler, Richard Houlston, María Soler Artigas, Kjell Grankvist, Mikael Johansson, Frances A. Shepherd, Michael W. Marcus, Hans Brunnström, Jonas Manjer, Olle Melander, David C. Muller, Kim Overvad, Antonia Trichopoulou, Rosario Tumino, Geoffrey Liu, Stig E. Bojesen, Xifeng Wu, Loic Le Marchand, Demetrios Albanes, Heike Bickeböller, Melinda C. Aldrich, William S. Bush, Adonina Tardon, Gad Rennert, M. Dawn Teare, John K. Field, Lambertus A. Kiemeney, Philip Lazarus, Aage Haugen, Stephen Lam, Matthew B. Schabath, Angeline S. Andrew, Pier Alberto Bertazzi, Angela C. Pesatori, David C. Christiani, Neil Caporaso, Mattias Johansson, James D. McKay, Paul Brennan, Rayjean J. Hung, Christopher I. Amos

**Affiliations:** ^1^ Baylor College of Medicine, Houston, TX, USA; ^2^ Laval University, Quebec, QC, Canada; ^3^ Department of Biomedical Data Science, Dartmouth College, Hanover, NH, USA; ^4^ University Health Network, The Princess Margaret Cancer Centre, Toronto, CA, USA; ^5^ Department of Oncology, Herlev and Gentofte Hospital, Copenhagen University Hospital, Copenhagen, Denmark; ^6^ Fred Hutchinson Cancer Research Center, Seattle, WA, USA; ^7^ Swedish Medical Group, Seattle, WA, USA; ^8^ Department of Oncology, University of Sheffield, Sheffield, UK; ^9^ Research Unit of Molecular Epidemiology, Institute of Epidemiology II, Helmholtz Zentrum München, German Research Center for Environmental Health, Neuherberg, Germany; ^10^ Thoraxklinik at University Hospital Heidelberg, Translational Lung Research Center Heidelberg (TLRC-H), Heidelberg, Germany; ^11^ Translational Lung Research Center Heidelberg (TLRC-H), Heidelberg, Germany; ^12^ German Center for Lung Research (DKFZ), Heidelberg, Germany; ^13^ University of Salzburg and Cancer Cluster, Salzburg, Austria; ^14^ Department of Genetic Epidemiology, University Medical Center, Georg-August-University Göttingen, Göttingen, Germany; ^15^ Indiana University, Bloomington, IN, USA; ^16^ University of Toronto, Toronto, ON, Canada; ^17^ University of Kentucky, Markey Cancer Center, Lexington, KY, USA; ^18^ Department of Thoracic Oncology, H. Lee Moffitt Cancer Center and Research Institute, Tampa, FL, USA; ^19^ Institute of Pneumology “Marius Nasta”, Bucharest, Romania; ^20^ Faculty of Medicine, University of Ostrava, Ostrava, Czech Republic; ^21^ Clinical Center of Serbia, School of Medicine, University of Belgrade, Belgrade, Serbia; ^22^ M. Sklodowska-Curie Cancer Center, Institute of Oncology, Warsaw, Poland; ^23^ Department of Epidemiology and Prevention, N.N. Blokhin Russian Cancer Research Center, Moscow, Russian Federation; ^24^ International Organization for Cancer Prevention and Research, Belgrade, Serbia; ^25^ Department of Surgery, National Tuberculosis and Lung Diseases Research Institute, Warsaw, Poland; ^26^ International Agency for Research on Cancer, World Health Organization, Lyon, France; ^27^ Nofer Institute of Occupational Medicine, Department of Environmental Epidemiology, Lodz, Poland; ^28^ Department of Clinical Science, University of Bergen, Bergen, Norway; ^29^ National Institute of Occupational Health, Oslo, Norway; ^30^ Unit of Nutrition and Cancer, Catalan Institute of Oncology (ICO-IDIBELL), Barcelona, Spain; ^31^ University of Pittsburgh Cancer Institute, Pittsburgh, PA, USA; ^32^ The Institute of Cancer Research, London, UK; ^33^ Department of Health Sciences, Genetic Epidemiology Group, University of Leicester, Leicester, UK; ^34^ National Institute for Health Research (NIHR) Leicester Respiratory Biomedical Research Unit, Glenfield Hospital, Leicester, UK; ^35^ Department of Medical Biosciences, Umeå University, Umeå, Sweden; ^36^ Department of Radiation Sciences, Umeå University, Umeå, Sweden; ^37^ Princess Margaret Cancer Centre, Toronto, ON, Canada; ^38^ Institute of Translational Medicine, University of Liverpool, Liverpool, UK; ^39^ Department of Pathology, Lund University, Lund, Sweden; ^40^ Faculty of Medicine, Lund University, Lund, Sweden; ^41^ School of Public Health, St. Mary’s Campus, Imperial College London, London, UK; ^42^ Section for Epidemiology, Department of Public Health, Aarhus University, Aarhus, Denmark; ^43^ Hellenic Health Foundation, Athens, Greece; ^44^ Molecular and Nutritional Epidemiology Unit CSPO (Cancer Research and Prevention Centre), Scientific Institute of Tuscany, Florence, Italy; ^45^ Lunenfeld-Tanenbaum Research Institute of Mount Sinai Hospital, University of Toronto, Toronto, Canada; ^46^ Department of Clinical Biochemistry, Herlev and Gentofte Hospital, Copenhagen University Hospital, Denmark; ^47^ Faculty of Health and Medical Sciences, University of Copenhagen, Copenhagen, Denmark; ^48^ Copenhagen General Population Study, Herlev and Gentofte Hospital, Copenhagen, Denmark; ^49^ Department of Epidemiology, The University of Texas MD Anderson Cancer Center, Houston, TX, USA; ^50^ Epidemiology Program, University of Hawaii Cancer Center, Honolulu, HI, USA; ^51^ Division of Cancer Epidemiology and Genetics, National Cancer Institute, National Institutes of Health, Bethesda, MD, USA; ^52^ Department of Thoracic Surgery, Division of Epidemiology, Vanderbilt University Medical Center, Nashville, TN, USA; ^53^ Department of Epidemiology and Biostatistics, School of Medicine, Case Western Reserve University, Cleveland, OH, USA; ^54^ IUOPA, University of Oviedo and CIBERESP, Faculty of Medicine, Campus del Cristo s/n, Oviedo, Spain; ^55^ Clalit National Cancer Control Center at Carmel Medical Center and Technion Faculty of Medicine, Haifa, Israel; ^56^ School of Health and Related Research, University of Sheffield, Sheffield, UK; ^57^ Radboud University Medical Center, Nijmegen, The Netherlands; ^58^ Department of Pharmaceutical Sciences, College of Pharmacy, Washington State University, Spokane, WA, USA; ^59^ National Institute of Occupational Health, Oslo, Norway; ^60^ British Columbia Cancer Agency, Vancouver, Canada; ^61^ Department of Cancer Epidemiology, H. Lee Moffitt Cancer Center and Research Institute, Tampa, FL, USA; ^62^ Department of Epidemiology, Geisel School of Medicine, Hanover, NH, USA; ^63^ Department of Preventive Medicine, IRCCS Foundation Ca’ Granda Ospedale Maggiore Policlinico, Milan, Italy; ^64^ Department of Clinical Sciences and Community Health, University of Milan, Milan, Italy; ^65^ Department of Epidemiology, Program in Molecular and Genetic Epidemiology Harvard School of Public Health, Boston, MA, USA; ^66^ Biomedical Data Science Department, Dartmouth College, Hanover, NH, USA

**Keywords:** epistasis, lung cancer, oncogenesis, functional annotation

## Abstract

The development of cancer is driven by the accumulation of many oncogenesis-related genetic alterations and tumorigenesis is triggered by complex networks of involved genes rather than independent actions. To explore the epistasis existing among oncogenesis-related genes in lung cancer development, we conducted pairwise genetic interaction analyses among 35,031 SNPs from 2027 oncogenesis-related genes. The genotypes from three independent genome-wide association studies including a total of 24,037 lung cancer patients and 20,401 healthy controls with Caucasian ancestry were analyzed in the study. Using a two-stage study design including discovery and replication studies, and stringent Bonferroni correction for multiple statistical analysis, we identified significant genetic interactions between SNPs in *RGL1:RAD51B* (OR=0.44, *p* value=3.27x10^-11^ in overall lung cancer and OR=0.41, *p* value=9.71x10^-11^ in non-small cell lung cancer), *SYNE1:RNF43* (OR=0.73, *p* value=1.01x10^-12^ in adenocarcinoma) and *FHIT:TSPAN8* (OR=1.82, *p* value=7.62x10^-11^ in squamous cell carcinoma) in our analysis. None of these genes have been identified from previous main effect association studies in lung cancer. Further eQTL gene expression analysis in lung tissues provided information supporting the functional role of the identified epistasis in lung tumorigenesis. Gene set enrichment analysis revealed potential pathways and gene networks underlying molecular mechanisms in overall lung cancer as well as histology subtypes development. Our results provide evidence that genetic interactions between oncogenesis-related genes play an important role in lung tumorigenesis and epistasis analysis, combined with functional annotation, provides a valuable tool for uncovering functional novel susceptibility genes that contribute to lung cancer development by interacting with other modifier genes.

## INTRODUCTION

Lung cancer, as one of the most common cancers worldwide, has a complex disease mechanism and both genetic and environmental factors, as well as the interactions among those factors contribute to development of this deadly disease [1-9]. The past decade has witnessed the harvest of genome-wide association studies (GWAS) in complex disease studies and several common variants predisposing to lung cancer have been identified including *TERT* at 5p15, *TP63* at 3q28, *HLA* region at 6p21, and *CHRNB4-CHRNA3-CHRNA5* region at 15q25, etc [2-6]. However, the discovered genetic variants only account for a limited fraction of the heritability of lung cancer [10]. Genetic interactions, i.e., epistasis is believed to contribute to a considerable proportion of the missing heritability in complex human diseases [11-12]. Epistasis, is the phenomenon where the effect of one gene is dependent on the effects of one or more other genes that may not be detected solely by studying the main effect of either gene alone. There is growing evidence showing that epistasis is involved in lung cancer development [9,13-14]. In 2014, researchers conducted a genome-wide gene-gene interaction analysis and identified an epistasis effect between rs2562796 (gene: *HIBCH*) and rs16832404 (gene: *c2orf88*) in lung cancer development [9]. Neither of these two genes has been identified from main effect association analysis before, suggesting that genetic interactions, especially those among novel variants, remain unrevealed in lung cancer study. To date, the reports on large-scale genetic interaction analysis in human diseases remain quite limited because of the challenge in high-dimensional data analysis.

The development of cancer is driven by the accumulation of many oncogenesis-related genetic alterations and tumorigenesis is triggered by complex networks of involved genes rather than independent actions [15-18]. Extensive molecular studies have revealed interactions existing between selected cancer driver genes, such as cooperation of *MYC* and *RAS* in transformation and immortalization process, and *BRCA1* and *P53* in breast cancer development, etc [19-20]. These are only tips of iceberg and much more latent genetic interactions among cancer-related genes are waiting to be identified. We hypothesized that there are considerable interactions among oncogenesis-related genes underlying lung tumorigenesis and a GxG interaction association analysis provides us a potent tool to explore it. An epistasis study among oncogenesis-related genes in lung cancer will help us identify oncogenes or tumor suppressors affecting early stages of lung cancer development that cannot be captured by single-locus analysis; provide insights about the connected pathways and genetic networks involved in lung cancer development; and discover novel targets for disease treatment. And the results from interaction analysis can be leveraged to improve lung cancer risk assessment.

Lung cancer is a heterogeneous disease and researchers have identified vast differences in genomic attributes, such as specific variants, gene mutation, gene expression and DNA methylation profile, etc., between adenocarcinoma (ADE) and squamous cell carcinoma (SQC) lung cancer subtype [4, 21-22]. However, the knowledge about epistatic features in lung cancer subtypes is limited. Performing a stratified epistasis analysis by lung cancer histology subtype will provide insights concerning tumor-subtype specific genetic interactions and gene networks. The availability of large lung cancer GWAS data from international collaboration enables us to conduct a large-scale epistasis analysis among oncogenesis-related genes in overall lung cancer as well as lung cancer subtypes. In this study, we collected the genotype data from 44,438 individuals with Caucasian ancestry, including 20,401 controls and 24,037 cases, from three independent cohorts. It is currently the largest genetic interaction analysis in lung cancer study to our knowledge.

## RESULTS

**Figure 1:**
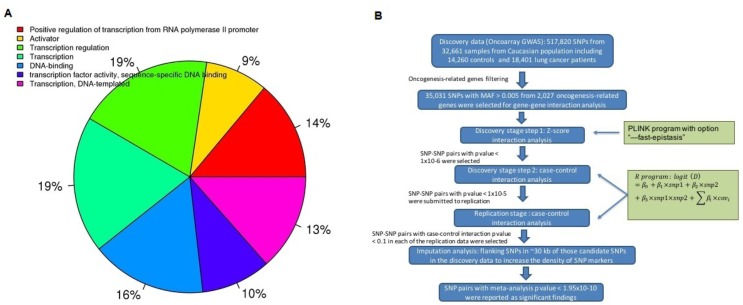
Carcinogenesis-related gene selection and statistical analysis strategy used in the study **A.** Categorization of the molecular functions of the selected 2027 cancer-related genes (DAVID). **B.** Flow chart of statistical analysis strategy in the study.

The genotypes from three independent lung cancer GWAS including a total of 24,037 lung cancer patients and 20,401 health controls with European ancestry were collected for the study. Demographic and clinical characteristics as well as sample sizes are summarized in Table 1. A comprehensive list of 2,027 cancer-related genes, including DNA binding proteins, transcription factors, transcriptional regulators, and other genes regulating protein expression, were identified and used to filter the search space for genetic interactions between carcinogenesis-related genes (Figure 1A). The study strategy is presented in Figure 1B. A stringent Bonferroni corrected significance cutoff was calculated based on the number of pair-wise tests between independent SNPs and *p* value < 1.95x10^-10^ was used for significance threshold in final meta-analysis.

**Table 1:** Summary and characteristics of three independent GWAS datasets used in the study

	OncoArray *N* = 32,661		Affymetrix *N* = 10,347		GELCC *N* = 1,430	
	Discovery		Replication 1		Replication 2	
No. Sample	Cases *n* = 18,401	Controls *n* = 14,260	Cases *n* = 4,950	Controls *n* = 5,397	Cases *n* = 686	Controls *n* = 744
Age^a^	63.8	61.6	62.9	60.4	61.6	64.9
Male (%)	62.5	60.3	53.9	53.4	38.9	35.6
Smoking status						
Never (%)	9.7	32.1	9.7	29.8	13.0	40.1
Former (%)	38.7	39.7	36.2	35.2	87.0^c^	59.9^c^
Current (%)	51.6	28.2	54.1	34.9	NA	NA
Packyr^b^	40.7	29.8	28.9	27.6	NA	NA
Histology						
NSCLC (%)	73.9		63.3		70.8	
ADE (%)	38.9		36.4		40.7	
SQC (%)	25.1		19.9		14.3	

a and b, average statistics for age and packyr (pack year) are provided. c, includes both current and former smokers.

### Discovery study

By use of the “fast-epistasis” option in PLINK to quickly screen for interactions, an exhaustive pairwise interaction analysis among cancer-related genes was conducted in the overall lung cancer cohort (ALL) as well as the stratified group by NSCLC, ADE and SQC histology subtype using lung cancer OncoArray GWAS data in the discovery stage. The sample size in the cohorts included 14,260 controls plus 18,401 overall lung cancer cases, 13,593 NSCLC cases, 7,157 ADE cases and 4,612 SQC cases, respectively. In the ALL interaction analysis, there were 102, 734, 7113 and 70145 SNP pairs when we used *p* value < 1x10^-7^, 1x10^-6^, 1x10^-5^ and 1x10^-4^ as the significance threshold, respectively. We decided to use 1x10^-6^ as the significance cutoff value to provide a balance between including excess false positive results *versus* missing potential signals. There were 717, 917 and 1118 SNP pairs with Z-score *p* value < 1x10^-6^ from NSCLC, ADE and SQC interaction analysis, respectively (Appendix 3 Supplementary Table 1). The selected SNP pairs were submitted to more stringent logistic regression interaction analysis. A total of 678, 673, 883 and 1062 SNP pairs had logistic interaction *p* value < 1x10^-5^ from ALL, NSCLC, ADE and SQC cohort, respectively. These SNP pairs were further submitted to replication study.

### Replication study

In the replication study, we first performed logistic gene-gene interaction studies for the selected SNP pairs in Affymetrix GWAS and GELCC GWAS datasets separately and then conducted meta-analysis to combine the information from both studies. 37, 50, 33 and 67 SNP pairs had a replication meta-analysis *p* value < 0.05 with consistent interaction effects (either risk or protective effect) across all three different datasets in ALL, NSCLC, ADE and SQC, respectively (Appendix 1). A final meta-analysis was conducted to combine the information from both discovery and replication studies at these replicated SNP pairs and overall ORs and *p* values were reported. Table 2 displays the results for SNP pairs with logistic interaction *p* value < 1x10^-5^ in discovery dataset and < 0.1 from each of the two replication datasets. In ALL lung cancer cohort, we identified a significant interaction effect between rs74826777, located in gene *RGL1*, and five SNPs located at gene *RAD51B*. The interaction OR varied from 0.46 to 0.49 with *p* values ranging from 1.14x10^-8^ to 2.83x10^-7^in discovery study. In the replication study, the interaction OR varied from 0.16 to 0.30 with *p* values ranging from 3.56x10^-4^ to 9.63x10^-3^in Affymetrix replication data; and OR varied from 0.20 to 0.34 with *p* value ranging from 2.00x10^-2^ to 8.62x10^-2^ in GELCC replication data. In the overall meta-analysis, three SNP pairs had *p* value < 1.95x10^-10^ (highlighted in red in Table 2). The most significant SNP pair was rs74826777:rs17835244 with an overall interaction OR of 0.44 and *p* value of 3.27x10^-11^. One of the SNP pairs, rs74826777:rs1474960, also displayed significant signals in NSCLC cohort with OR 0.40 and *p* value 9.71x10^-11^ in final meta-analysis.

**Table 2:** Signals from interaction analysis in genotyped discovery data, replication data sets and joint analysis

						OncoArray_genotyped		Affymetrix_imputed		GELCC_imputed		Joint		
SNP1	A1 (MAF)	GENE1	SNP2	A1 (MAF)	GENE2	OR	P	OR	P	OR	P	OR	P	Q
rs74826777	A (0.01)	RGL1	rs4902626	A (0.21)	RAD51B	0.49	2.83E-07	0.30	9.63E-03	0.22	2.68E-02	0.46	3.24E-09	0.32
rs74826777	A (0.01)	RGL1	rs2877496	A (0.21)	RAD51B	0.47	5.20E-08	0.18	6.71E-04	0.24	4.35E-02	0.43	1.59E-10	0.14
rs74826777	A (0.01)	RGL1	rs1474960	G (0.22)	RAD51B	0.48	1.39E-07	0.16	3.56E-04	0.20	2.00E-02	0.43	3.11E-10	0.06
rs74826777	A (0.01)	RGL1	rs17835218	A (0.22)	RAD51B	0.46	1.14E-08	0.17	4.66E-04	0.34	8.62E-02	0.42	6.60E-11	0.15
rs74826777	A (0.01)	RGL1	rs17835244	C (0.22)	RAD51B	0.48	2.89E-08	0.17	4.84E-04	0.29	5.66E-02	0.44	3.27E-11	0.11
rs3764240	A (0.03)	CD109	rs851984	A (0.39)	ESR1	1.36	1.63E-06	1.41	9.61E-03	2.08	1.85E-02	1.39	1.01E-09	0.41
rs3764240	A (0.03)	CD109	rs851983	G (0.39)	ESR1	1.37	9.29E-07	1.41	9.84E-03	2.08	1.81E-02	1.39	1.01E-09	0.41
rs3764240	A (0.03)	CD109	rs851982	G (0.39)	ESR1	1.37	9.86E-07	1.43	7.36E-03	2.08	1.81E-02	1.39	8.30E-10	0.41
rs7783961	A (0.28)	CALCR	rs2505532	A (0.41)	RET	1.14	4.08E-07	1.10	9.38E-02	1.26	6.03E-02	1.14	8.30E-07	0.62
NSCLC														
rs74826777	A (0.01)	RGL1	rs2877496	A (0.21)	RAD51B	0.45	1.56E-07	0.16	1.68E-03	0.24	6.20E-02	0.41	3.81E-10	0.18
rs74826777	A (0.01)	RGL1	rs1474960	G (0.22)	RAD51B	0.44	7.23E-08	0.13	6.84E-04	0.22	3.92E-02	0.40	9.71E-11	0.11
rs9677398	A (0.28)	THADA	rs2648875	A (0.24)	PVT1	1.17	4.41E-07	1.11	8.62E-02	1.34	7.20E-02	1.16	9.50E-09	0.46
rs7570751	G (0.28)	THADA	rs2648875	A (0.24)	PVT1	1.17	3.07E-07	1.11	8.44E-02	1.47	1.87E-02	1.17	5.46E-09	0.24
rs6544655	G (0.28)	THADA	rs2648875	A (0.24)	PVT1	1.17	6.05E-07	1.11	7.29E-02	1.39	4.36E-02	1.17	4.79E-09	0.41
rs6544657	G (0.28)	THADA	rs2648875	A (0.24)	PVT1	1.17	2.93E-07	1.11	8.42E-02	1.38	4.56E-02	1.17	7.91E-09	0.38
rs1554783	G (0.25)	SYNE1	rs10515157	A (0.16)	RNF43	0.84	1.78E-06	0.81	3.55E-03	0.69	3.70E-02	0.82	1.29E-08	0.57
ADE														
rs2131556	A (0.21)	PTPRU	rs4646	A (0.27)	CYP19A1	0.82	8.54E-07	0.87	8.79E-02	0.64	2.48E-02	0.82	2.70E-08	0.37
rs1554783	G (0.25)	SYNE1	rs10515157	A (0.16)	RNF43	0.79	3.04E-07	0.79	8.69E-03	0.52	5.47E-03	0.78	3.18E-09	0.22
rs2758791	G (0.26)	SYNE1	rs10515157	A (0.16)	RNF43	0.79	2.31E-07	0.79	8.68E-03	0.58	1.48E-02	0.78	4.28E-09	0.38
SQC														
rs6716971	G (0.06)	BRE	rs6787614	A (0.12)	RUVBL1	1.63	1.12E-06	2.12	2.07E-04	5.30	8.92E-02	1.74	6.03E-10	0.26
rs1882898	A (0.38)	FHIT	rs1705235	C (0.05)	TSPAN8	1.51	1.45E-07	2.26	2.91E-02	9.95	9.31E-04	1.57	5.95E-09	0.01
rs11135724	G (0.27)	LOXL2	rs208311	G (0.30)	P2RX7	0.81	6.63E-07	0.82	3.76E-02	0.58	6.03E-02	0.81	6.31E-09	0.52

There are 37, 50, 33 and 67 SNP pairs with meta-analysis *p* value < 0.05 in replication study and only SNP pairs with interaction *p* value < 0.1 in both the two replication datasets are reported in Table 2. OR, *p* values from each individual dataset as well as the joint meta-analysis are reported, and numbers highlighted in red color indicate the overall meta-analysis *p* values < 1.95x10^-10^ (Bonferroni corrected significance cutoff).

In addition to the significant interaction effect detected between *RGL1* and *RAD51B*, we also identified some interactions with consistent evidence across discovery and replication studies but not achieving significance threshold (*p* value < 1.95x10^-10^) in the overall meta-analysis, such as *CD109*:*ESR1* gene pair in ALL cohort, *THADA:PVT1* in NSCLC cohort, *SYNE1*:*RNF43* from ADE cohort, and *BRE*:*RUVBL1* and *FHIT*:*TSPAN8* from SQC cohort (Table 2). Suggestive evidence for genetic interactions between *SYNPO2* and *BRCA1* gene were found in ADE cohort. 8 SNP pairs had significant interaction effect in the OncoArray discovery dataset and GELCC replication study. However, no supporting evidence was identified in Affymetrix replication data (Appendix 1). The most significant signal came from rs6828669:rs16941 with overall OR of 0.85 and *p* value of 3.95x10^-9^.

### Imputation analysis in candidate regions

**Figure 2:**
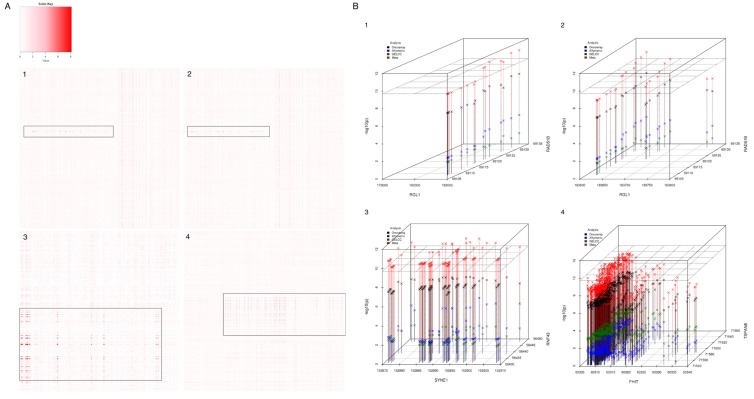
Imputed genetic interaction analysis in candidate regions 1-4 plots display the results at all lung cancer, NSCLC, ADE and SQC cohort, respectively. **A.** Interaction map with SNPs from ∼ 30 kb flanking regions using imputed genotype at discovery dataset. The X and Y axis denote the SNPs at each of the gene. The color shade indicates the change of -log10(p) of interaction *p* value. The signals were highlighted in grey-colored boxes. **B.** Signals from analysis using imputed genotype. The X and Y axis denote location (bp) of each of the SNP in one gene and Z axis displayed the -log10(p) from the interaction analysis, the plane in dash line indicated *p* value of 1.95x10-10. Black, blue and green color denotes the results from OncoArray, Affymetrix and GELCC imputed genotype data analysis and red color denotes the results from joint analysis combining all the datasets.

For each unique gene pair reported in Table 2, we further imputed the ∼30kb flanking regions harboring the involved SNPs using the genotypes from the OncoArray GWAS data to increase the density of markers in the candidate regions. Figure 2A displayed the interaction mapping using imputed genotype in discovery data. In each plot, X and Y-axis denote the imputed SNPs in each of the gene and -log10(p) was represented by shades of red color in the heatmap. More potential significant SNPs pairs were revealed in the intensive interaction mapping and highlighted in grey-colored boxes. For example, 16 and 25 SNP pairs, between *RGL1* and *RAD51B* gene, were found with interaction *p* value < 1x10^-6^ in ALL and NSCLC discovery cohort, respectively (Appendix 2). We further validated the signals in replication datasets and performed joint analysis to combine all information. Figure 2B displayed the significant signals from the imputed genotype analysis. In each plot, X and Y axis denoted the location of SNPs at each of the gene and Z axis displayed the -log10(p) in the joint meta-analysis, the plane in dash line indicated Bonferroni corrected *p* value threshold (1.95x10^-10^). For the genetic interaction between *RGL1* and *RAD51B* gene, we identified 7 and 3 SNP pairs with joint *p* value < 1.95x10^-10^ from the ALL lung cancer and NSCLC cohort, respectively. The most significant SNP pair in ALL cohort is rs74826777:rs8006890 with an OR of 0.39 and *p* value of 1.68x10^-11^; and rs74826777:rs17835244 with OR 0.38 and *p* value of 3.76x10^-11^ in NSCLC cohort (Figure 2B 1&2, Appendix 2). The interaction analysis using imputed genotype data reinforced the finding for interaction between *RGL1* and *RAD51B* gene.

In the ADE and SQC cohort, we identified some evidence for genetic interactions in *SYNE1:RNF43* and *FHIT:TSPAN8* gene pairs in discovery and replication study but none of the SNP pairs achieved the significance level (Table 2). Fortunately, the intensive interaction mapping using imputed genotype provided us strong evidence for epistasis between these two gene pairs. In ADE subtype, we identified 111 SNP pairs with meta-analysis *p* value < 1.95x10^-10^ coming from 14 SNPs at *SYNE1* and 8 SNPs at *RNF43* gene. The most significant interaction came from SNP pair rs12213593:rs11079348 with OR 0.73 and *p* value of 1.01x10^-12^. In SQC cohort, 21 SNP pairs, coming from 16 SNPs at *FHIT* and 3 SNPs at *TSPAN8*, had meta-analysis *p* value < 1.95x10^-10^. The most significant SNP pair was rs1882898:rs1798081 with OR 0.60 and *p* value of 7.62x10^-11^ (Figure 2B 3&4, Appendix 2).

### Epistasis in lung cancer risk development

**Figure 3:**
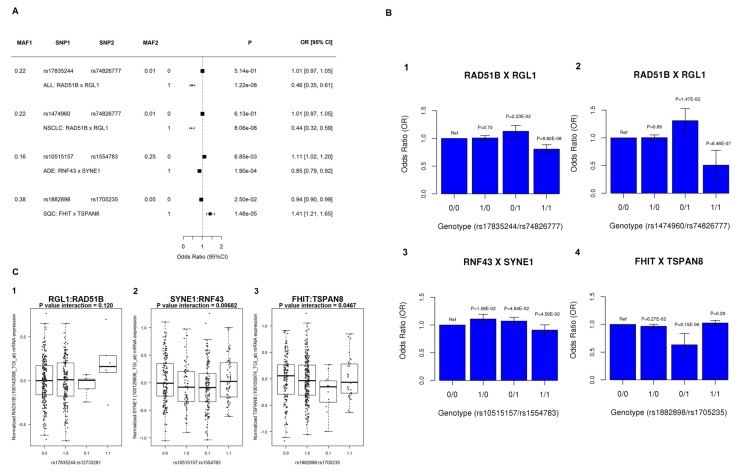
Interaction analysis at candidate gene pairs **A.** Stratified lung cancer risk analysis at the identified significant SNP pairs using genotype data in discovery OncoArray data. MAF1 and MAF2 indicate the minor allele frequency of each SNP in the pair. 0 and 1 indicate the genotype without or with at least one copy of minor allele in second SNP. *P* values and ORs of SNP1 in main effect association conditioned on genotype at SNP2 were displayed. **B.** the bar plots of risk effect at joint genotypes. In each plot, the genotype with no MAF at either locus (0/0) was used as reference group. **C.** Analysis of the genetic interaction effect on gene expression in human lung tissues. The interaction effects of SNP pairs on gene expression levels were evaluated using linear models. The evaluated genes and selected probes were labeled at Y-axes. The boxplots of gene expression level were plotted for each joint genotype group to display the genetic interaction effect on gene expression. Genotypes were coded in a dominant mode, i.e. 0 or 1 for absence or presence of the minor allele, respectively.

In order to investigate how the genetic variation at one locus impacted the risk effect at the other locus in the identified significant SNP pairs we further conducted stratified association analysis. Take the rs74826777:rs17835244 pair from *RGL1:RAD51B* gene pair as an example, these two SNPs had *p* value of 0.16 and 0.02 in single-locus association analysis in ALL lung cancer cohort, respectively, indicating no significant main effect in lung cancer development. In the stratified analysis, for individuals with no minor allele at rs74826777 (*RGL1* gene), rs17835244 (*RAD51B*) does not impact lung cancer risk (*p* value = 0.51); but for individuals with at least one copy of minor alleles at rs74826777, rs17835244 displays a protective effect for lung cancer with OR 0.46 and *p* value of 1.22x10^-8^ (Figure 3A). This association of rs17835244 with lung cancer is so significant that it achieves a genome-wide significance level in a standard GWAS study (*p* < 5x10^-8^). Similar results were detected in *RAD51B:RGL1* gene pair in NSCLC cohort (Figure 3A). These results present a perfect example explaining epistasis contributes to lung cancer risk development which cannot be revealed by single-locus main effect screening.

For the *RNF43:SYNE1* gene pair identified in ADE cohort, rs10515157 slightly increases the ADE risk (OR = 1.11 and *p* value = 6.85x10^-3^) among individuals carrying no minor allele at rs1554783; whereas decreases ADE risk among individuals carrying at least one minor allele at rs1554783 (OR = 0.85 and *p* value = 1.90x10^-4^). For the *FHIT:TSPAN8* gene pair identified in SQC cohort, rs1882898, located in *FHIT*, slightly decreases SQC risk among individuals with no minor allele at rs1705235 (OR = 0.94 and *p* value = 2.50x10^-2^); whereas increases SQC risk among individuals with at least one minor allele at rs1705235 (OR = 1.41 and *p* value = 1.48x10^-5^). These results displayed reverse risk effect at one genetic locus when the genotype was different at second locus.

**Figure 4:**
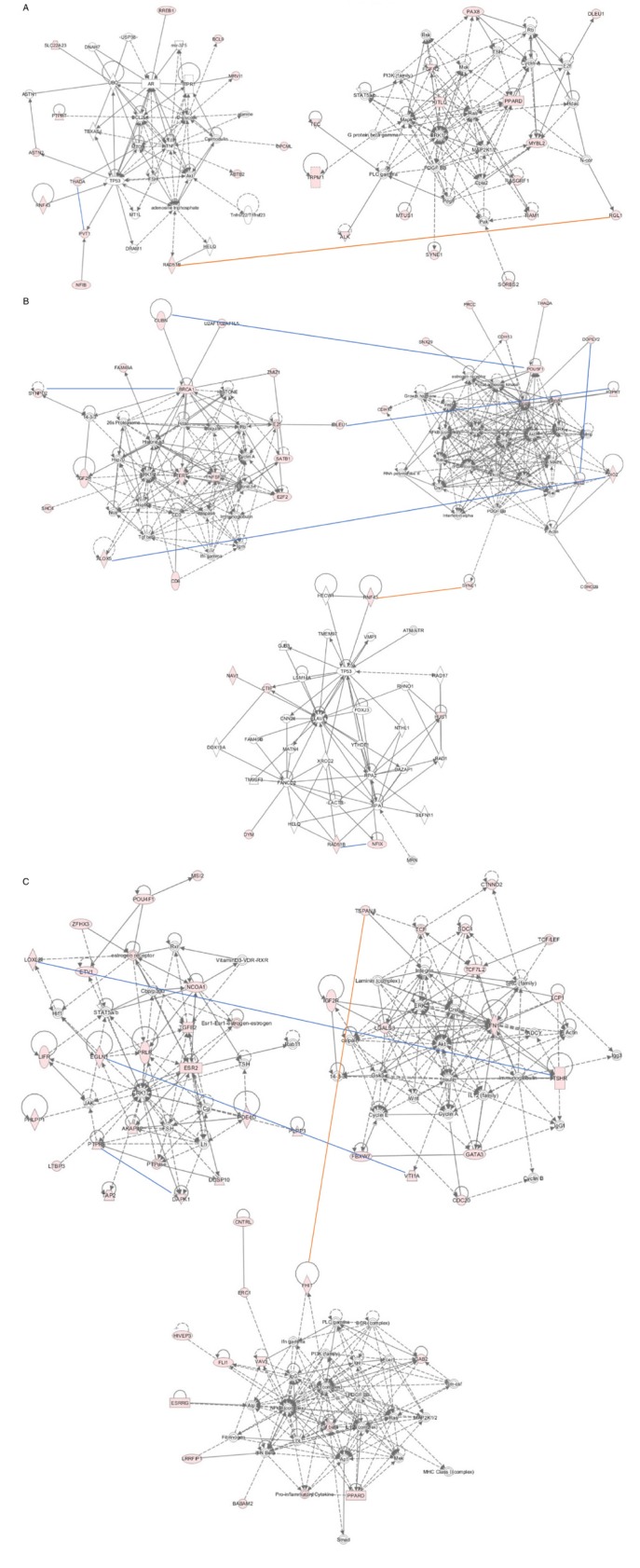
Gene network analysis using IPA program **A.**-**C.** displayed the top networks from NSCLC, ADE and SQC cohort, respectively. Genes labeled with pink color indicate the input candidate genes from interaction analysis. Arrow lines indicate the potential genetic interactions identified in G x G analysis. Red lines indicate those genetic interactions either achieving the significance level in the joint analysis or having multiple SNP pairs with consistent evidence for genetic interaction across discovery and replication data sets although not achieving significance level in joint analysis; blue line indicate sporadic signals for potential genetic interactions without achieving significance.

We further analyzed the lung cancer risk effect with joint genotypes at the SNP pairs, with no minor allele at either locus (0/0) as reference group. For SNP pair rs74826777:rs1474960, from *RGL1:RAD51B* gene pair, individuals with genotype 1/0 had a lung cancer risk effect with OR 1.31 and *p* value 0.01 whereas individuals with genotype 1/1 had a reduced effect with OR 0.51 and *p* value 6.46x10^-7^ (Figure 3B-2). Similar effect was obtained in all lung cancer cohort (Figure 3B-1). For *FHIT:TSPAN8* gene pair identified from SQC cohort, individuals with genotype 0/1 had a significant reduced lung cancer risk with OR 0.63 and *p* value 9.15x10^-6^ (Figure 3B-4).

### Genetic interactions on gene expression in the lung

We further explored the impact of identified genetic interactions on the expression of involved cancer-related genes using the lung eQTL dataset including a total of 409 subjects with both genotyping and gene expression data [23-24]. We evaluated the interaction effects of the significant SNP pairs on gene expression levels. For the significant SNP pair rs10515157:rs1554783 from *RNF43:SYNE1* gene pair, *SYNE1* gene expression levels are significantly different across four groups with different joint genotypes (Figure 3C-2, *P* = 6.82x10^-3^). With a dominant genotype model in both of the SNPs, the individuals with 1/0 and 0/1 joint genotype have lower *SYNE1* expression compared with 0/0 and 1/1 groups. Another differentially expressed gene is *TSPAN8* from SNP pair rs1882898:rs1705235 (Figure 3C-3, *P* = 4.67x10^-2^). We detected decreased gene expression in 1/0, 0/1 and 1/1 groups compared with 0/0 genotype group. For the SNP pair rs17835244:rs74826777 from *RGL1*:*RAD51B* gene pair, the rs74826777 is not available in the lung eQTL dataset and was replaced by the best available proxy (rs12733281, D’ = 1, R^2^ = 0.33 in European population). Because the low allele frequency in rs74826777 (and proxy rs12733281) (MAF ∼ 0.01), we had a limited number of samples to test the interaction. We did not identify differential gene expression in the gene pairs, but we did see a trend of increased expression in 1/1 genotype group (Figure 3C-1, *P* = 0.12). No significant interaction signals were found for expression levels of *RGL1*, *RNF43*, and *FHIT* (Appendix 3 Supplementary Figure 1).

### Gene set enrichment analysis

The statistical analysis using GWAS data provided very significant statistical evidence for genetic interactions between selected genes. However, we wanted to further explore the underlying biological mechanisms behind the statistical findings and understand the genetic architecture of epistasis acting more generally. In ALL lung cancer cohort, 37 SNP pairs had meta-analysis *p* value < 0.05 in replication analysis and displayed consistent interaction effect across three independent datasets. These 37 SNP pairs came from 38 unique cancer-related genes and these genes were submitted to IPA program for pathway and network analysis. Similarly, 43, 36 and 76 genes were submitted to IPA program from NSCLC, ADE and SQC cohorts, respectively. The top 5 canonical pathways from each lung cancer histology subtype are listed in Table 3 and all of them had a Fisher exact test *p* value less than 0.01 suggesting the input genes were biologically connected rather than randomly associated. The pathway “hot spot” genes from ALL and NSCLC are *EGF, FGFR2, EGFR*, and *GSK3B*, etc. The regulation of the epithelial-mesenchymal transition (EMT) pathway is among the top 5 canonical pathways in both ALL (*p* = 1.79x10^-5^) and NSCLC (*p* = 6.02x10^-3^) lung cancer cohorts. EMT is an evolutionary conserved process which is induced in the metastasis process, converting stationary epithelial cells to invasive and mobile mesenchymal cells [25-28]. The top 2 canonical pathways in ADE cohort, both include *BRCA1* and *HUS1* gene, are Role of CHK Proteins in Cell Cycle Checkpoint Control (*p* = 1.12x10^-4^) and DNA damage-induced 14-3-3σ Signaling pathway (*p* = 4.40x10^-4^). Both these two pathways have been reported to be involved in lung carcinogenesis and prognosis [29-31].

**Table 3:** Top 5 canonical pathways involving the genes from identified genetic interactions in each lung cancer subtype

Subset	Canonical pathways	*P*	Overlap
ALL	Glioblastoma Multiforme Signaling (TSC1, EGF, PLCB1, FGFR2, GSK3B, EGFR)	3.35x10-7	6/162
	HER-2 signaling (TSC1, EGF, FGFR2, GSK3B, EGFR)	4.18x10-7	5/88
	Gαq Signaling (CALCR, NFATC2, PLCB1, FGFR2, GSK3B)	8.24x10-6	5/161
	Regulation of the Epithelial-Mesenchymal Transition Pathway (NOTCH4, EGF, FGFR2, GSK3B, EGFR)	1.79x10-5	5/189
	ErbB Signaling (EGF, FGFR2, GSK3B, EGFR)	2.46x10-5	4/98
NSCLC	Regulation of the Epithelial-Mesenchymal Transition Pathway (BCL9, EGFR, FGFR2)	6.02x10-3	3/189
	UVB-Induced MAPK Signaling (EGFR, FGFR2)	7.43x10-3	2/66
	EGF Signaling (EGFR, FGFR2)	7.87x10-3	2/68
	Caveolar-mediated Endocytosis Signaling (EGFR, ITGA11)	8.55x10-3	2/71
	ErbB4 Signaling (FGFR2, YAP1)	8.79x10-3	2/71
ADE	Role of CHK Proteins in Cell Cycle Checkpoint Control (BRCA1, E2F2, HUS1)	1.12x10-4	3/57
	DNA damage-induced 14-3-3σ Signaling (BRCA1, HUS1)	4.40x10-4	2/19
	Glioma Signaling (E2F2, EGFR, IGF2R)	8.60x10-4	3/114
	Role of Oct4 in Mammalian Embryonic Stem Cell Pluripotency (BRCA1, POU5F1)	2.59x10-3	2/46
	Spliceosomal Cycle (U2AF1/U2AF1L5)	3.28x10-3	1/2
SQC	Osteoarthritis Pathway (CASP8, FN1, GLI3, PaRX7, PPARD, SDC4, TCF7L2)	8.84x10-6	7/212
	Protein Kinase A Signaling (AKAP12, CDC25C, DUSP10, GLI3, PDE4D, PLCB1, PTPRE, TCF7L2, TGFB2)	1.00x10-5	9/401
	Aryl Hydrocarbon Receptor Signaling (ATR, ESR1, ESR2, TGFB2)	1.46x10-3	4/141
	Inflammasome Pathway (CASP8, P2RX7)	2.16x10-3	2/20
	Molecular Mechanisms of Cancer (ATR, CASP8, CDC25C, GAB2, PLCB1, TGFB2)	2.45x10-3	6/394

Fisher exact test p value is displayed to evaluate if the input genes are biologically connected rather than randomly associated. Overlap indicates the number of input genes overlapped with the number of genes in a well-known canonical pathway.

In addition to the already known canonical pathways, we are also interested in the *de novo* gene networks that may exist among the interactive genes. Figure 4A-4C plots displayed the top putative gene networks curated by epistasis-involved candidate genes from NSCLC, ADE and SQC subgroup interaction analysis. In each plot, red lines indicate genetic interactions either achieving the significance level (*p* < 1.95x10^-10^) in the joint meta-analysis; blue lines indicate SNP pairs with consistent evidence for genetic interaction across discovery and replication data sets but not achieving significance level in joint analysis. In NSCLC lung cancer cohort, IPA created two top gene networks with *RAD51B* in one network and *RGL1* in the other (Figure 4A). Similar gene networks results were found in ALL lung cancer cohort (Appendix 3 Supplementary Figure 2). The significant genetic interactions between *SYNE1* and *RNF43* in the ADE cohort, and between *TSPAN8* and *FHIT* in SQC cohort were both demonstrated as interactions between two putative networks (Figure 4B-4C). In addition to the significant between-network interactions in *RAD51B:RGL1*, *SYNE1:RNF43* and *TSPAN8:FHIT* pairs, we also see within-network interactions, such as the interaction between *THADA* and *PVT1* in the NSCLC cohort, and between *BRCA1* and *SYNPO2* in ADE cohort, etc (Figure 4A-4B).

## DISCUSSION

Epistasis is an important mechanism contributing to development of complex human diseases. However, new discoveries of genetic interactions derived from association analysis have been limited because of the challenge in high-dimensional statistical analysis. In this paper, we reported a filtered G x G interaction analysis among oncogenesis-related genes in lung cancer development aiming to identify important oncogene or tumor suppressor genes involved in lung cancer development by interacting with other modifier genes. This study design also has the advantage to reduce the search space and thus improve the statistical power in epistasis analysis. We collected the GWAS data from lung cancer OncoArray Consortium in discovery stage and two independent GWAS data sets for replication study. A total of 44,438 individuals including 24,037 lung cancer patients and 20,401 health controls from European descent population were recruited in this study, which is by far the largest genetic interaction study in lung cancer to our knowledge. We successfully identified epistasis in *RGL1*:*RAD51B* in ALL and NSCLC lung cancer, *SYNE1:RNF43* in ADE and *FHIT:TSPAN8* in SQC risk development. None of the SNPs, from the significant SNP pairs, were revealed previously in main effect association analysis either from our discovery dataset (P > 0.01) or from other published lung cancer GWAS reports. These findings reinforce that genetic epistasis among cancer-related genes is a common mechanism involved in lung tumorigenesis and many latent genes contribute to lung cancer development through interacting with other modifier genes.

We further extended the lung cancer risk analysis to lung eQTL gene expression analysis. *SYNE1* was implicated in many cancers and gene expression profiles analysis in TCGA displayed that *SYNE1* was downregulated in 18 cancer types, including NSCLC, breast cancer and colon cancer, etc, compared with normal tissues [32]. In the gene expression analysis between *SYNE1* and rs10515157:rs1554783 SNP pair, the *SYNE1* gene expression were slightly decreased in groups with 1/0 and 0/1 joint genotype and increased in 1/1 group compared with 0/0 reference group (Figure 3C-2). These results were consistent with the observed increased lung cancer risk in 1/0 and 0/1 group, and decreased risk in 1/1 group in our epistasis analysis (Figure 3B-3). In the study between *TSPAN8* gene and rs1882898:rs1705235 SNP pair, we also identified consistent evidence between risk and gene expression analysis. *TSPAN8* gene played an important role in cancer initiation and overexpression was discovered in colorectal, pancreatic and NSCLC, etc [33-35]. The decreased expression in joint genotype 1/0 and 0/1 group were consistent with the decreased lung cancer risk in these two groups. *RAD51B*, as a tumor suppressor gene, belongs to *RAD51* protein family that is essential for DNA repair by homologous recombination. Overexpression of *RAD51B* is found to be associated with better prognosis in NSCLC [36-37]. For the *RAD51B* gene, we did not identify interaction signals in gene expression analysis because of the limited sample size to test low frequency alleles and there were only 6 individuals with the highest risk joint genotype (Figure 3C-1). However, we see a trend of increased expression in 1/1 group and decreased expression in 0/1 group, which supports the decreased risk effect in 1/1 and increased risk in 0/1 group, respectively (Figure 3B 1-2). In summary, the gene expression analysis provides information about the functional role of the identified genetic interactions in lung cancer development, supporting the findings of this study and yield insights about the molecular mechanisms of lung carcinogenesis.

In the gene network analysis, the IPA program constructed gene networks by searching the extensive records maintained in its library to find the genes that may directly or indirectly connected with the input “seeds” from interaction analysis. Interestingly, all the three significant epistasis—-*RGL1*:*RAD51B* in ALL and NSCLC lung cancer, *SYNE1:RNF43* in ADE and *FHIT:TSPAN8* in SQC risk development—-were displayed as interaction between networks (Figure 4 and Appendix 3 Supplementary Figure 2). These results suggest that lung cancer has a very complex molecular mechanism and interactions among gene networks, rather than just interactions between individual genes, are involved in lung cancer development.

Lung cancer is a heterogeneous disease and different genetic variants have been identified associated with overall lung cancer risk as well as lung cancer subtypes [4,38]. The large sample size in our study and the high density of imputed SNP markers allow us to identify lung cancer subtype-specific epistasis including *RGL1:RAD51B* in NSCLC (OR = 0.40, *p* = 9.71x10^-11^), *SYNE1*:*RNF43* in ADE cohort (OR = 0.73, *p* = 1.01x10^-12^), and *FHIT*:*TSPAN8* in SQC cohort (OR = 0.60, *p* = 7.62x10^-11^). The pathway and gene network analysis also display the differences in epistasis and signaling pathways in lung cancer subtype which enhances our understanding of the molecular mechanisms underlying lung cancer subtypes.

Stringent Bonferroni correction, assuming the independence among all the tests, was applied to control the multiple comparison issue in our study. However, the Bonferroni multiple test correction is overly conservative for pair-wise interaction analysis. The pair-wise interaction tests are positively correlated with each other. Our application of the overly conservative Bonferroni corrected *p* value < 1.95x10^-10^ may have led to our not detecting some significant findings. We believe more genetic interactions including those with small effects and histology subtype-specific effects could be identified in the future as more samples with genotype data become available. With current knowledge, the information about the functional significance of the identified SNPs is remains limited in our study, but we were fortunate to be able to analyze effects on joint genotypes from a study of lung tissues. With the development of the functional annotations on the GWAS SNP panel we wish to identify genetic interactions with important diagnosis and prognosis value in lung cancer disease in the future.

## MATERIALS AND METHODS

### Study populations

We collected the genotype data from three independent lung cancer GWAS including a total of 24,037 lung cancer patients and 20,401 health controls with Caucasian ancestry in the study. TRICL (Transdisciplinary Research In Cancer of the Lung) OncoArray consortium GWAS data were analyzed in the discovery stage, including 14,260 controls and 18,401 lung cancer patients (dbGaP Study Accession: phs001273.v1.p1) (Table 1)^4^. All the samples were genotyped using the Illumina OncoArray-500K BeadChip and 502,933 SNPs remained for analysis after quality control processes. Two independent lung cancer GWAS data were analyzed in the replication study: TRICL Affymetrix GWAS data including 5,397 controls and 4,950 lung cancer cases (http://www.ncbi.nlm.nih.gov/projects/gap/cgi-bin/study.cgi?study_id=phs000876.v1.p1), and GELCC (Genetic Epidemiology of Lung Cancer Consortium) familial lung cancer GWAS including 744 controls and 686 lung cancer patients genotyped using Illumina HumanOmniExpressExome-8v1 array [38-39]. The demographic and clinical characteristics of each dataset including age, gender, and smoking status, histology subtypes, etc., were provided in Table 1. IBD analysis was conducted between the datasets and duplicated samples were removed before the epistasis analysis.

### Ethics statement

All subjects provided informed consent, and the institutional review boards of each participating institutes approved this collaborative study. Further details about the specific studies are provided in prior studies of the Oncoarray, TRICL Affymetrix array, and GELCC study [4,38-39]. Data from all of these studies have been uploaded to dbGAP (phs001273.v1.p1, phs0 phs000878.v1.p1).

### Cancer related genes filtering process

We sought to obtain a list including the oncogenesis-related gene as complete as possible for this filtered epistasis analysis. The Bushman Lab generated a comprehensive list of 2,027 cancer-related genes which were selected based on information from the Atlas of Genetics and Cytogenetics in Oncology and Hematology, Catalog of Somatic Mutations in Cancer (COSMIC), and the Consensus Coding Sequences of Human Breast and Colorectal Cancer, etc [40]. The majority of these genes are encoded for DNA binding proteins, transcription factors, transcriptional regulators, and other genes regulating protein expression (Figure 1A) [41]. The well-known lung cancer related genes, such as lung tumor mutation-harboring genes *EGFR, KRAS, BRAF* and risk-associated genes - nicotinic acetylcholine receptor family (*CHRNB4, CHRNA3, CHRNA5*, etc.), CYP gene family (*CYP1A1, CYP2A6, CYP2D6*, etc.), and *TERT*, etc., are also included in these 2,027 genes. The ∼500,000 post-quality control SNPs from the discovery OncoArray GWAS data were narrowed down to 43,652 SNPs located within transcript region (including untranslated regions) of these 2,027 cancer-related genes. We further removed SNPs with minor allele frequency (MAF) < 0.005 because these variants had little power in genetic interaction analysis and the number of SNPs in final analysis was 35,031.

### Statistical analysis

#### Significance *p* value threshold calculation

Specifying a reasonable Bonferroni corrected *p* value threshold is an important step in analysis of high dimensional data. Among the 35,031 tested SNPs, some of them are not independent from each other because of linkage disequilibrium (LD). We applied the GEC (Genetic Type I error calculator) program to evaluate the eigenvectors of the correlation matrix and computed the number of independent SNPs in the analysis [42]. We found there were 22,622 independent SNPs among the 35,031 tested SNPs and the number of pairwise interaction tests was 255,866,131. The Bonferroni corrected *p* value cutoff assuming independence among all the tests was computed as 0.05/255,866,131 = 1.95x10^-10^. Any SNP pairs with joint interaction *p* value < 1.95x10^-10^ will be reported as significant findings.

### Epistasis analysis

In this proposed genetic interaction analysis, we followed a two-stage study design: we conducted the interaction analysis at discovery stage using OncoArray GWAS data, and then replicated the signals using independent Affymetrix and GELCC GWAS datasets. A two-step analysis strategy was adopted in the discovery stage: in step 1, an imprecise but fast pairwise epistasis analysis was conducted using the “fast-epistasis” option in PLINK program [43]. This test was based on a Z-score test to compare the difference of SNP1-SNP2 allelic association between cases and controls. SNP pairs with *p* value < 1x10^-6^ were submitted to a more stringent logistic regression analysis using formula (1) at step 2. An R program was used for logistic regression interaction analysis and the first three principal components (PCs) were adjusted in the analysis. In order to harvest as many potential signals as possible in discovery study we relaxed the significance cutoff to 1x10^-5^ in regression analysis. The SNP pairs with logistic regression interaction *p* value less than 1x10^-5^ were further submitted to replication studies.

$$\mathrm{logit}\left(D\right)=\beta_{0}+\beta_{1}\times snp1+\beta_{2}\times snp2+\beta_{3}\times snp1\times snp2+\sum \beta_{i}\;x\;{\mathrm{cov}}_{i}$$ (1)

In the replication study, the same regression model specified by formula (1) was applied in the analysis. Considering the small sample size in the GELCC GWAS dataset (*n* = 1430) compared with the TRICL Affymetrix GWAS (*n* = 10,347), we performed a meta-analysis in the replication study to combine the information from both datasets. The SNP pairs with interaction meta-analysis *p* value < 0.05 and consistent interaction effects (OR > or < 1 across different discovery and replication datasets) were reported as replicated signals. We then performed a final meta-analysis to combine the information from all three datasets in discovery and replication stage. Figure 1B displayed the flowchart of research strategy in the epistasis study. We applied the same research strategy in overall lung cancer cohort as well as stratified analysis by lung cancer subtypes NSCLC (non-small cell lung cancer), ADE and SQC.

### Genotype imputation

The genotypes from the three GWAS datasets came from different platforms and the overlaps among the SNP panels were limited. We used IMPUTE2 program to impute the genotype in replication datasets to increase the SNP overlaps between the discovery and replication datasets. For those candidate SNP pairs with meta-analysis *p* value < 0.1 in each replication study we further imputed the flanking SNPs in ∼30 kb of those candidate SNPs in the discovery data to increase the density of SNP markers. The 1000 Genomes Project Phase 3 release was used as the reference dataset [44]. The output dosage file from IMPUTE2 was used as input in logistic regression analysis and the first three PCs were adjusted in the imputed genotype analysis.

### Genetic interactions on gene expression in the lung

Identified epistasis on lung cancer risk were extended to gene expression levels using the lung eQTL dataset [23-24]. Briefly, lung specimens were collected from patients undergoing lung cancer surgery and stored at the biobank of the “Institut universitaire de cardiologie et de pneumologie de Québec” (IUCPQ). Genotyping was carried out using the Illumina Human1M-Duo BeadChip. Expression profiling was performed using an Affymetrix custom array (see GEO platform GPL10379). A total of 409 subjects passed genotyping and gene expression quality controls. Expression values were adjusted for age, sex and smoking status. Probe sets and SNPs implicated in the identified genetic interactions for lung cancer risk were selected including *RGL1*:*RAD51B*, *RNF43*:*SYNE1*, and *FHIT*:*TSPAN8*. The interaction effects of SNP pairs on gene expression levels were evaluated using linear models. Genotypes were coded in a dominant mode, i.e. 0 or 1 for absence or presence of the minor allele, respectively.

### Gene set enrichment analysis

Gene set enrichment analysis provided information about the biological implications underlying statistical findings in our genetic interaction analysis. We conducted canonical pathway and gene network analysis using Ingenuity Pathway Analysis (IPA, QIAGEN Inc., https://www.qiagenbioinformatics.com/products/ingenuity-pathway-analysis) software to explore the possible relationships among the candidate genes. The oncogenesis-related genes from the SNP pairs with interaction meta-analysis *p* value < 0.05 in replication study were provided as the focus genes in IPA. IPA searched the extensive records maintained in its Ingenuity Pathways Knowledge Base (IPKB) and created the canonical pathway based on well-known biological and molecular pathways. In gene network analysis, the IPA program utilized the provided list of focus gene as “seeds” and searched IPKB to find the genes that may directly or indirectly connected with the “seeds” and then created a gene network. The IPA program will also conduct a Fisher exact test to compute the probability that the association between a given gene set and a given pathway, based on well-established signaling and metabolic pathways in IPA library, is due to random chance and provide a *p* value for the test.

## SUPPLEMENTARY MATERIALS FIGURES AND TABLE


