# Supplementary material for: Genetic interaction analysis among oncogenesis-related genes revealed novel genes and networks in lung cancer development
Source: Oncotarget. 2019 Mar 5;10(19):1760–74. doi: 10.18632/oncotarget.26678 (PMC6442994; doi:10.18632/oncotarget.26678)
Supplement: Supplementary file 1 [file oncotarget-10-1760-s001.pdf]

# Genetic interaction analysis among oncogenesis-related genes revealed novel genes and networks in lung cancer development

## SUPPLEMENTARY MATERIALS

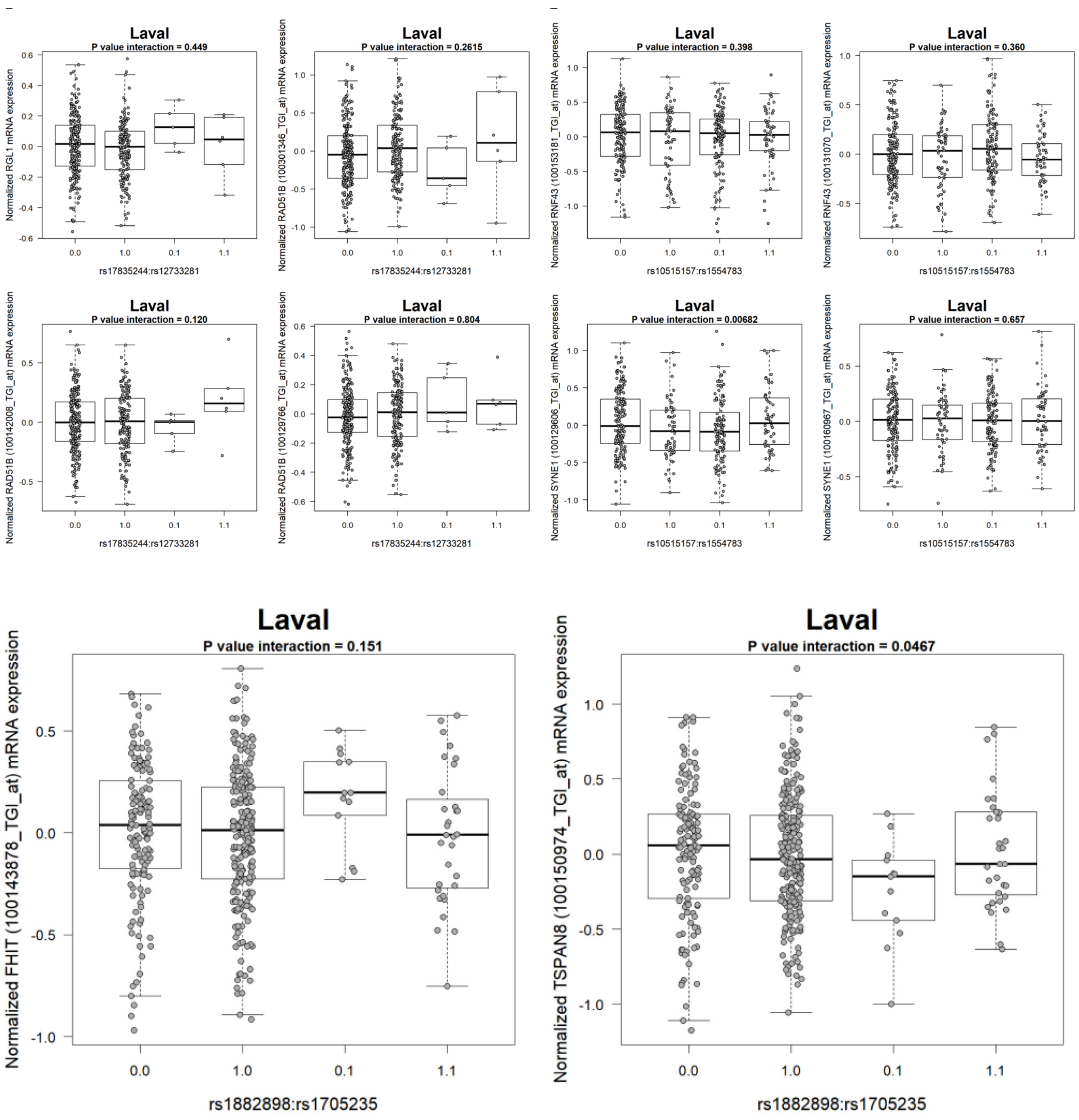

**Supplementary Figure 1: Analysis of genetic interaction effect on gene expression in human lung tissues.** Probe sets and SNPs implicated in the identified genetic interactions for lung cancer risk were selected including *RGL1*:*RAD51B* (S2-1), *RNF43*:*SYNE1* (S2-2), and *FHIT*:*TSPAN8* (S2-3). The interaction effects of SNP pairs on gene expression levels were evaluated using linear models. The evaluated genes and selected probes were labeled at Y-axes. The boxplots of gene expression level were plotted for each joint genotype group to display the genetic interaction effect on gene expression. Genotypes were coded in a dominant mode, i.e. 0 or 1 for absence or presence of the minor allele, respectively.

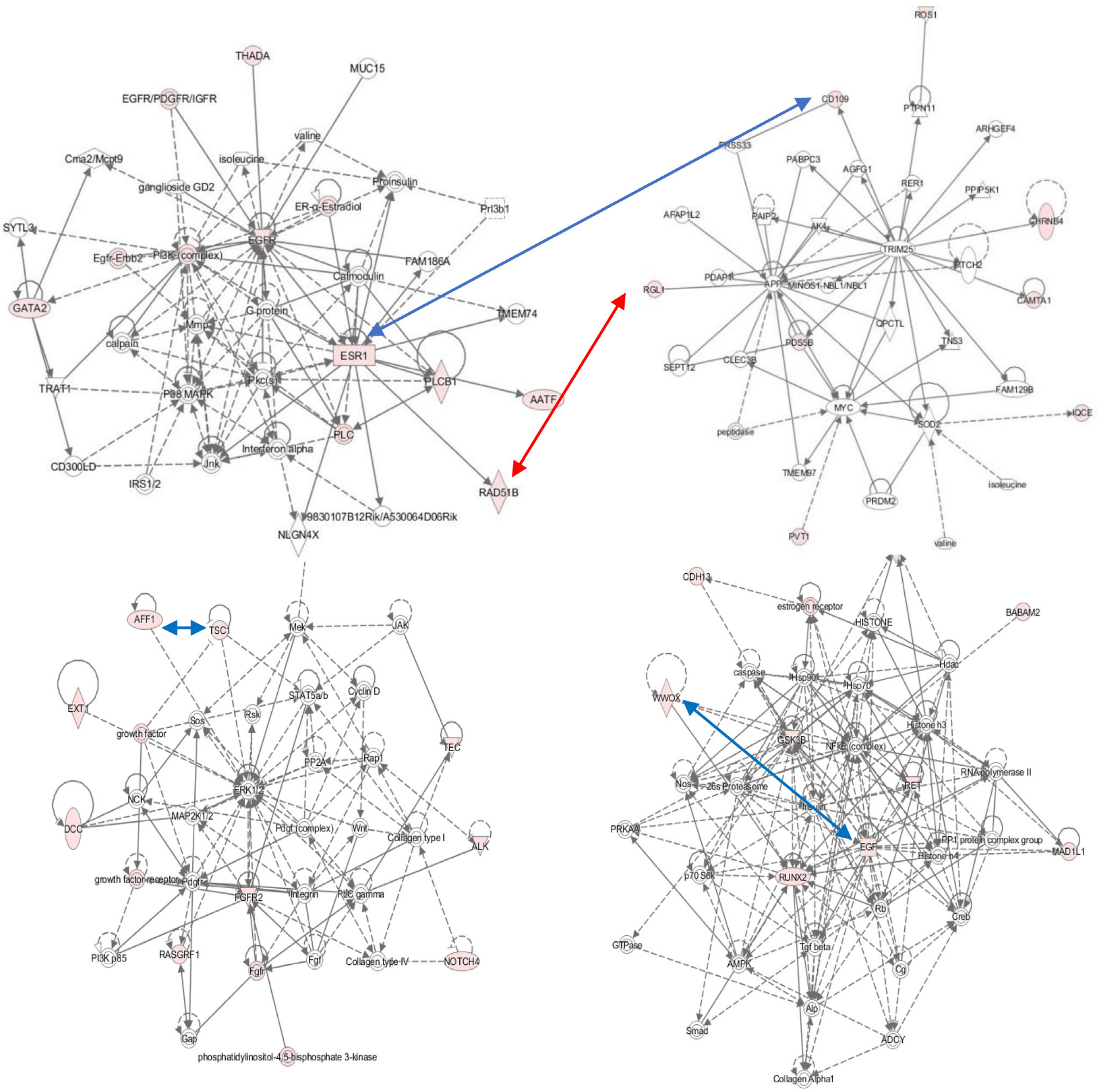


**Supplementary Figure 2: Top gene networks created by IPA program for ALL lung cancer cohort.**

**Supplementary Table 1: Number of SNP pairs with different p value cutoff in Z-score test at discovery stage**

| Study | $1x10^{-7}$ | $1x10^{-6}$ | $1x10^{-5}$ | $1x10^{-4}$ |
|---|---|---|---|---|
| ALL | 102 | 734 | 7113 | 70145 |
| NSCLC | 87 | 717 | 7137 | 69089 |
| ADE | 138 | 971 | 7459 | 70170 |
| SQC | 161 | 1118 | 7860 | 69089 |
